# Changes in the gene expression of co-cultured human fibroblast cells and osteosarcoma cells: the role of microenvironment

**DOI:** 10.18632/oncotarget.4902

**Published:** 2015-09-21

**Authors:** Viviana Salvatore, Stefano Focaroli, Gabriella Teti, Antonio Mazzotti, Mirella Falconi

**Affiliations:** ^1^ Department of Biomedical and Neuromotor Sciences, DIBINEM - University of Bologna, 40126, Bologna, Italy; ^2^ Rizzoli Orthopaedhic Institute, 40136 Bologna, Italy

**Keywords:** tumor microenvironment, YKL-40, inflammation, siRNA, osteosarcoma cells

## Abstract

**Background:**

The progression of malignant tumors does not depend exclusively on the autonomous properties of cancer cells; it is also influenced by tumor stroma reactivity and is under strict microenvironmental control. By themselves, stromal cells are not malignant, and they maintain normal tissue structure and function. However, through intercellular interactions or by paracrine secretions from cancer cells, normal stromal cells acquire abnormal phenotypes that sustain cancer cell growth and tumor progression. In their dysfunctional state, fibroblast and immune cells produce chemokines and growth factors that stimulate cancer cell growth and invasion. In our previous work, we established an *in vitro* model based on a monolayer co-culture system of healthy human fibroblasts (HFs) and human osteosarcoma cells (the MG-63 cell line) that simulates the microenvironment of tumor cells and healthy cells. The coexistence between MG-63 cells and HFs allowed us to identify the YKL-40 protein as the main marker for verifying the influence of tumor cells grown in contact with healthy cells.

**Methods:**

In this study, we evaluated the interactions of HFs and MG-63 cells in a transwell co-culture system over 24 h, 48 h, 72 h, and 96 h. We analyzed the contributions of these populations to the tumor microenvironment during cancer progression, as measured by multiple markers. We examined the effect of siRNA knockdown of *YKL-40* by tracking the subsequent changes in gene expression within the co-culture. We validated the expression of several genes, focusing on those involved in cancer cell invasion, inflammatory responses, and angiogenesis: *TNF alpha*, *IL-6*, *MMP-1*, *MMP-9*, and *VEGF*. We compared the results to those from a transwell co-culture without the *YKL-40* knockdown.

**Results:**

In a pro-inflammatory environment promoted by TNF alpha and IL-6, siRNA knockdown of *YKL-40* caused a down-regulation of *VEGF* and *MMP-1* expression in HFs.

**Conclusions:**

These findings demonstrated that the tumor microenvironment has an influence on the gene expression of healthy surrounding tissues and on the process of tumorigenicity and it is emerging as attractive targets for therapeutic strategies.

## INTRODUCTION

The constituents of the tumor microenvironment can arrive from two major avenues: recruitment from local tissue and systemic recruitment from distant tissues via circulation. The most accessible option for tumor cells engaged in stromal cell recruitment is to exploit the resources in close proximity to the site of tumor development. Tumor cells may alter the surrounding stroma through direct cell contact or by secreting paracrine-soluble factors, inducing cell modification and extracellular matrix (ECM) alterations [1]. In this context, inflammation and infection have received special attention; in fact, many inflammatory mediators may influence cell proliferation and tumor development, and they are responsible for invasion and immunosuppressive signaling through the production of angiogenic and growth factors, chemokines, cytokines, and matrix metalloproteinases [2].

The expression of various immune mediators and modulators, as well as the abundance and activation state of different cell types in the tumor microenvironment, lead to a tumor-promoting condition. Since tumor cells have a genetically-unstable background, the various factors and cytokines released by such cells might lead to or maintain an altered chronic wound-healing environment that further perpetuates a reactive stroma, leading to enhanced tumor progression and even metastasis [3]. In this study we focused on some key factors involved in tumor induction and their role in the ECM, angiogenesis, invasion, and metastasis. The effects of YKL-40 siRNA knockdown analyzing the mRNA of TNF alpha, IL-6, MMP-1, MMP-9 and VEGF were shown.

### TNF alpha

Tumor necrosis factor alpha (TNF-α) is a member of the TNF/TNFR cytokine superfamily. In common with other family members, TNF-α is involved in the maintenance and homeostasis of the immune system, inflammation, and host defense. It is an acute-response cytokine, but it is also involved in pathological processes such as chronic inflammation, autoimmunity, and in apparent contradiction to its name, malignant disease [4]. Cancer cell or stromal cell production of TNF-α is involved in the development of a range of tumors; it is partially responsible for NF-κB activation in initiated tumor cells and for the cytokine network found in human cancer. Via NF-κB, TNF-α is also central to the interactions between tumor cells and macrophages that result in not only increased invasive capacity of malignant cells, but also in the switch to an alternative tumor-promoting phenotype [5]. Increased serum concentrations of TNF-α are present in several independent cancer types, despite malignant cells constitutively producing only small amounts of TNF: TNF-α produced chronically at low picogram levels in the tumor microenvironment, whether by tumor or stromal cells (or most likely both), may cause direct DNA damage, have anti-apoptotic or mitogenic activity, mediate tumor/stromal cell interactions, and induce a range of MMPs, cytokines, and chemokines that promote tumor development. In established tumors, TNF-α contributes to the maintenance of a pro-inflammatory environment [6].

### IL-6

Another effect of TNF-α is the rapid induction of interleukin-6 (IL-6) gene expression particularly in monocytes and macrophages [7]. IL-6 is a glycoprotein and a multifunctional cytokine that, in addition to its more well-known roles as a pro-inflammatory and sclerotic agent, affects the activity of cancer cells. In parallel with the identification of its physiologic functions in orchestrating innate and adaptive immunity, IL-6 has emerged as a critical mediator for perpetuating chronic inflammation and autoimmunity, and it is increasingly recognized as a key cytokine for linking chronic inflammation to cancer development. Like TNF-α, IL-6 facilitates tumor development by promoting the conversion of non-cancer cells into tumor stem cells in low-attachment culture conditions by up-regulating *Oct 4* gene expression through activating the IL-6R/JAK/STAT3 signaling pathway [8]. The levels of IL-6 are elevated in advanced cancer, and elevated levels in human serum are associated with an increased risk of cancer. Because of that, IL-6 has been characterized as a prognostic marker of cancer [9].

### YKL-40

Human cartilage glycoprotein-39 (YKL-40) is a secreted glycoprotein originally identified in the medium of a human osteosarcoma cell line, MG-63. It is a highly phylogenetically conserved chitin-binding glycoprotein in the family of chitinase-like proteins. The biophysiologic activity of YKL-40 is poorly understood, but it is believed to be associated with the proliferation of connective tissue cells and the activation of vascular endothelial cells. YKL-40 purified from the MG-63 osteosarcoma cell line has growth factor activity in fibroblast cell lines [10]. YKL-40 secreted by cancer cells has a role in mutating the fibroblasts surrounding the tumor, causing the activation of fibroblast morphologic transformation, secretion of MMPs, and neovascularization. Therefore, YLK-40 promotes the proliferation, differentiation, and invasion of cancer cells and the destruction of stroma [11–13]. Serum levels of YKL-40 are elevated in a variety of chronic inflammatory diseases, suggesting that its pathologic function is connected with the process of ECM remodeling. The expression of YKL-40 is regulated by various cytokines and hormones, including IL-6 and TNF-α [14]. YKL-40 also enhances the contact of the tumor with the ECM, restricts vascular leakage, and stabilizes vascular networks [15].

### VEGF

The angiogenic switch, which occurs when a tumor begins growing vasculature, is determined by the imbalance between pro- and anti-angiogenic factors in the tumor microenvironment, which are directly secreted by tumor cells and indirectly secreted by cells in the microenvironment (perhaps induced by the tumor). Angiogenesis in tumor tissue is under the control of various factors released by tumor and stromal cells. VEGF is thought to be one of the most important determinants of angiogenesis in cancer; a high concentration of VEGF may induce aggressive tumor growth and metastasis [21]. Recently, YKL-40 has been discovered as a potent inducer of angiogenesis, and it has been investigated in several types of cancer. Francescone et al. elucidated the regulatory role of YKL-40 in VEGF production in glioblastoma cell line U87, and demonstrated how blocking YKL-40 activity with monoclonal antibodies is a promising therapeutic strategy for advanced tumors [22].

### MMP-1 and MMP-9

The expression of various MMPs is up-regulated in virtually every type of human cancer and correlates with advanced stage, invasive and metastatic properties, and in general, poor prognosis [16, 17]. The early expression of MMPs, either by tumor cells or surrounding stromal cells, helps to remodel the ECM and release ECM- and/or membrane-bound growth factors, which provide a favorable microenvironment for the establishment of the primary tumor [18]. Both MMP-1 and MMP-9 are up-regulated by TNF-α and are implicated in the induction of the angiogenic switch in different model systems [19, 20]. Further up-regulation of MMP expression, in particular the gelatinases, which can degrade basement membrane components, allows the tumor cells to invade the adjacent stroma and to break down the basement membranes associated with capillaries and lymphatic vessels, allowing tumor cells to enter circulation. MMPs are also involved in cell migration by removing sites of adhesion, exposing new binding sites, cleaving cell-cell or cell-matrix receptors, and releasing chemoattractants from the ECM [18]. Similar to intravasation, MMPs are necessary for circulating tumor cells to exit blood vessels (extravasation), although this step does not appear to be rate limiting for the establishment of metastases. At the distant site, MMPs are required for local migration, establishment of a microenvironment conducive to metastatic growth, and angiogenesis for sustained growth. Thus, MMPs contribute to the carcinogenic process at multiple stages [18].

## RESULTS

Co-cultured human fibroblasts (HFs) and MG-63 cells with silenced *YKL-40* had differences in gene expression levels compared to those of control co-cultured HFs and MG-63 cells. Below, we describe the results by gene.

### *TNF-α* expression

**Figure 1:**
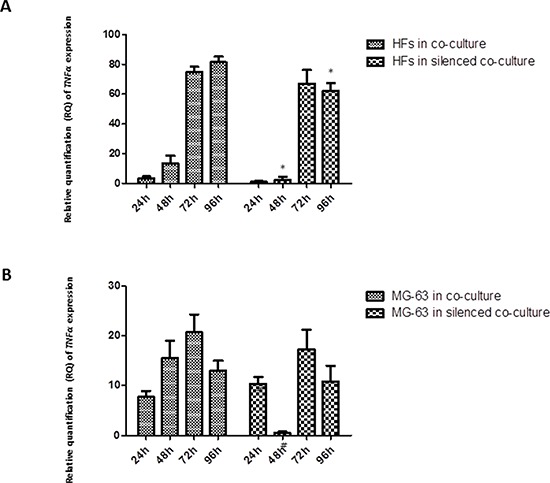
A. and B Real-Time PCR for *TNF-α*. The gene expression levels of *TNF-α* had a similar trend in co-culture and co-culture with silenced *YKL-40*: *TNF-α* mRNA increased significantly in HFs, reaching the highest level at 72 h and 96 h, whereas in the tumor cells, it remained constant and low. * represents a significant difference from HF cells grown in normal co-culture, *P* < 0.05. # represents a significant difference from MG-63 cells grown in normal co-culture, *P* < 0.05.

As assessed by a real-time PCR assay, the concentration of *TNF-α* mRNA increased significantly in HFs upon co-culturing, whereas its mRNA concentration in tumor cells remained low and constant. A similar trend was observed in co-cultured HFs with *YKL-40*-silenced MG-63 cells: an increased amount of *TNF-α* mRNA in HFs and a constant but low concentration in MG-63 cells (Figure 1).

### *IL-6* expression

**Figure 2:**
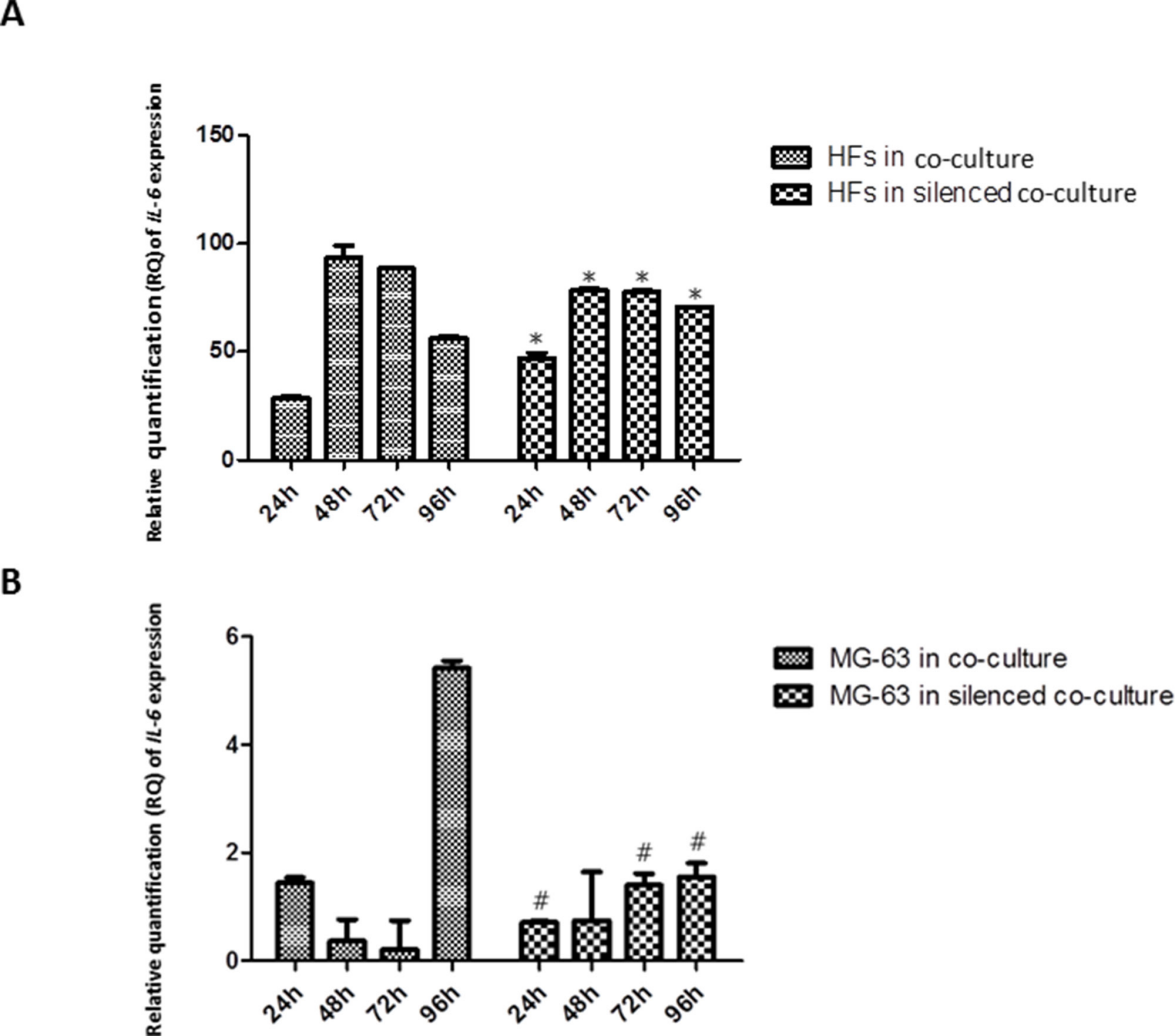
A. and B Real-Time PCR for *IL-6. IL-6* mRNA had high expression in HFs from co-culture and co-culture with silenced *YKL-40*. MG-63 cells in both co-culture conditions poorly expressed *IL-6*. * represents a significant difference from HF cells grown in normal co-culture, *P* < 0.05. # represents a significant difference from MG-63 cells grown in normal co-culture, *P* < 0.05.

*IL-6* expression in HFs increased over the experimental time points, reaching its highest concentration at 72 h in co-culture with MG-63 cells. A lower, but constant expression was found in HFs derived from the silenced co-culture. By contrast, MG-63 cells expressed very low levels of *IL-6*, both under normal co-culture conditions and in the silenced condition (Figure 2).

### *YKL-40* expression

**Figure 3:**
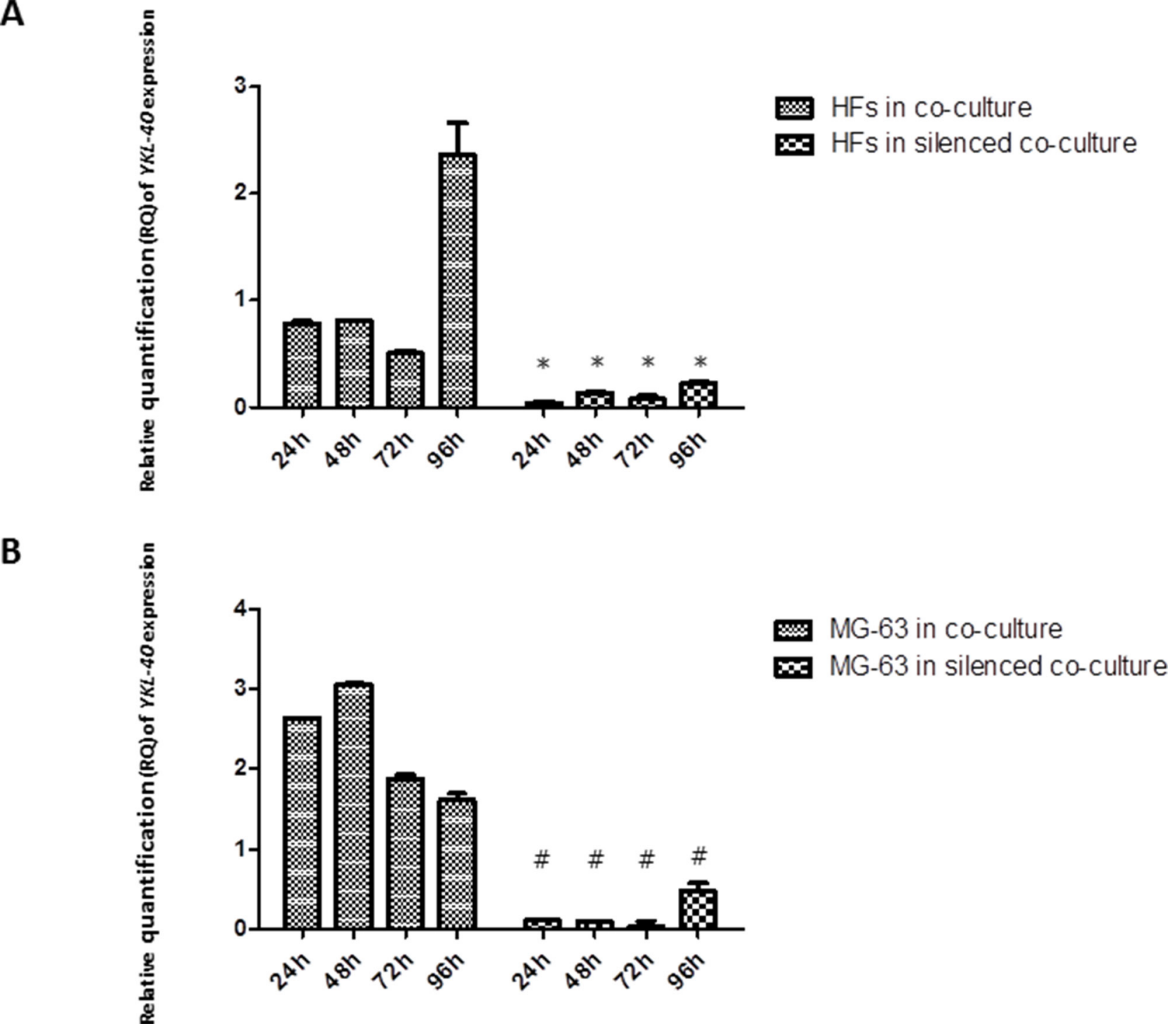
A. and B Real-Time PCR for *YKL-40*. In MG-63 cells, siRNA knockdown of *YKL-40* reached the maximum efficiency at 72 h, with respect to the expression of *YKL-40* in normally co-cultured MG-63 cells, in which the levels of *YKL-40* are significantly higher. In normal co-culture, fibroblasts had increased levels of *YKL-40*, reaching the highest peak at 96 h. In fibroblasts co-cultured with the silenced MG-63 cells, *YKL-40* was at the minimum level. * represents a significant difference from HF cells grown in normal co-culture, *P* < 0.05. # represents a significant difference from MG-63 cells grown in normal co-culture, *P* < 0.05.

siRNA knockdown of *YKL-40* reached the maximum efficiency at 72 h in MG-63 cells, as compared to the expression of *YKL-40* in normally co-cultured MG-63 cells, in which it was strongly expressed. In normal co-culture, fibroblasts had increased levels of *YKL-40*, reaching the highest peak at 96 h. *YKL-40* was at low levels in fibroblasts co-cultured with the silenced MG-63 cells (Figure 3).

### *VEGF* expression

**Figure 4:**
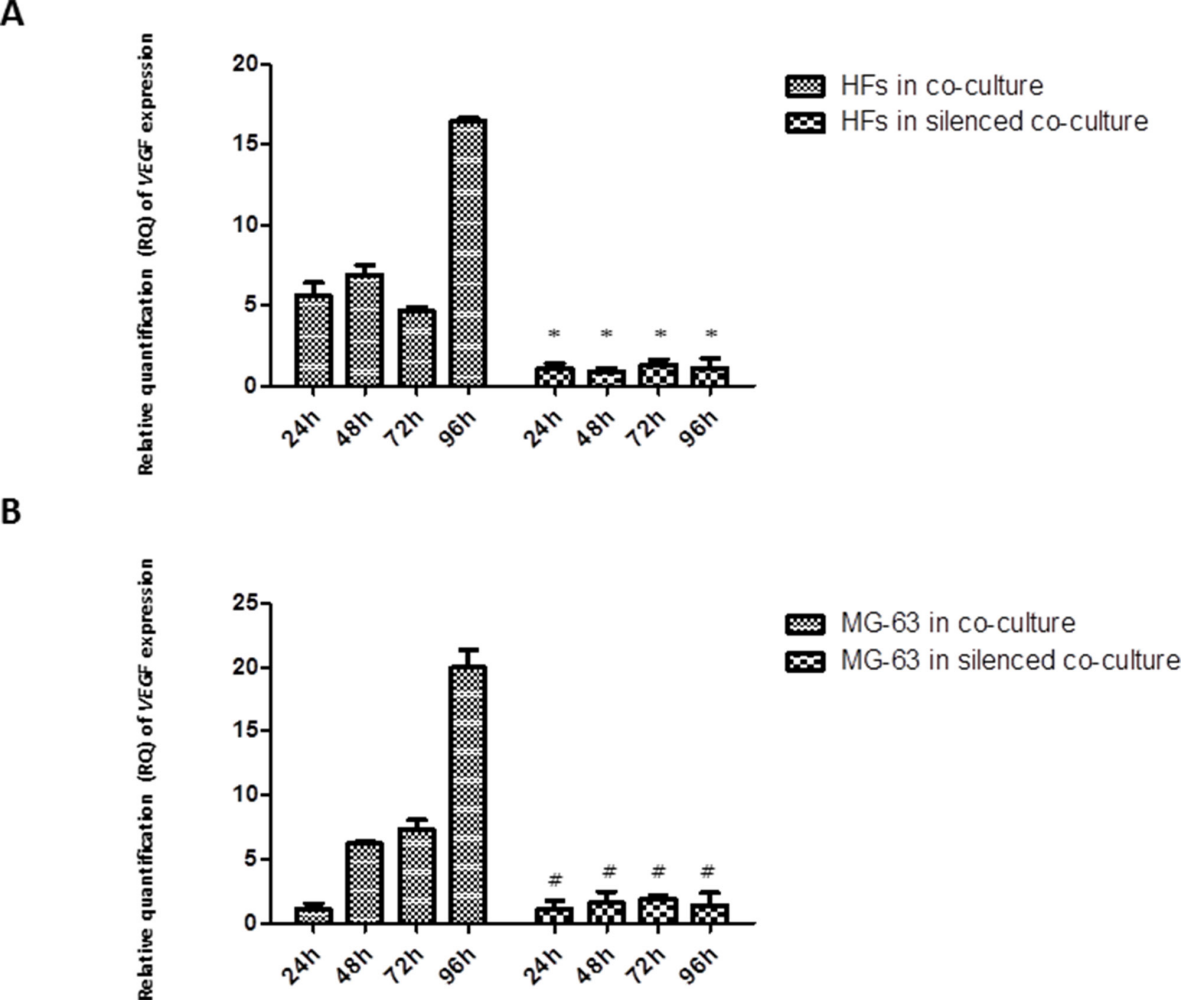
A. and B Real-Time PCR for *VEGF. VEGF* expression in fibroblasts and MG-63 cells in normal co-culture continued to increase through 96 h. In the *YKL-40*-silenced co-culture, the levels of *VEGF* were low in both cell populations. * represents a significant difference from HF cells grown in normal co-culture, *P* < 0.05. # represents a significant difference from MG-63 cells grown in normal co-culture, *P* < 0.05.

*VEGF* had similar expression levels in HFs and MG-63 cells grown in normal co-culture, reaching the highest expression at 96 h, whereas in both HFs and MG-63 cells grown in silenced co-culture, the expression was low (Figure 4).

### *MMP* expression

**Figure 5:**
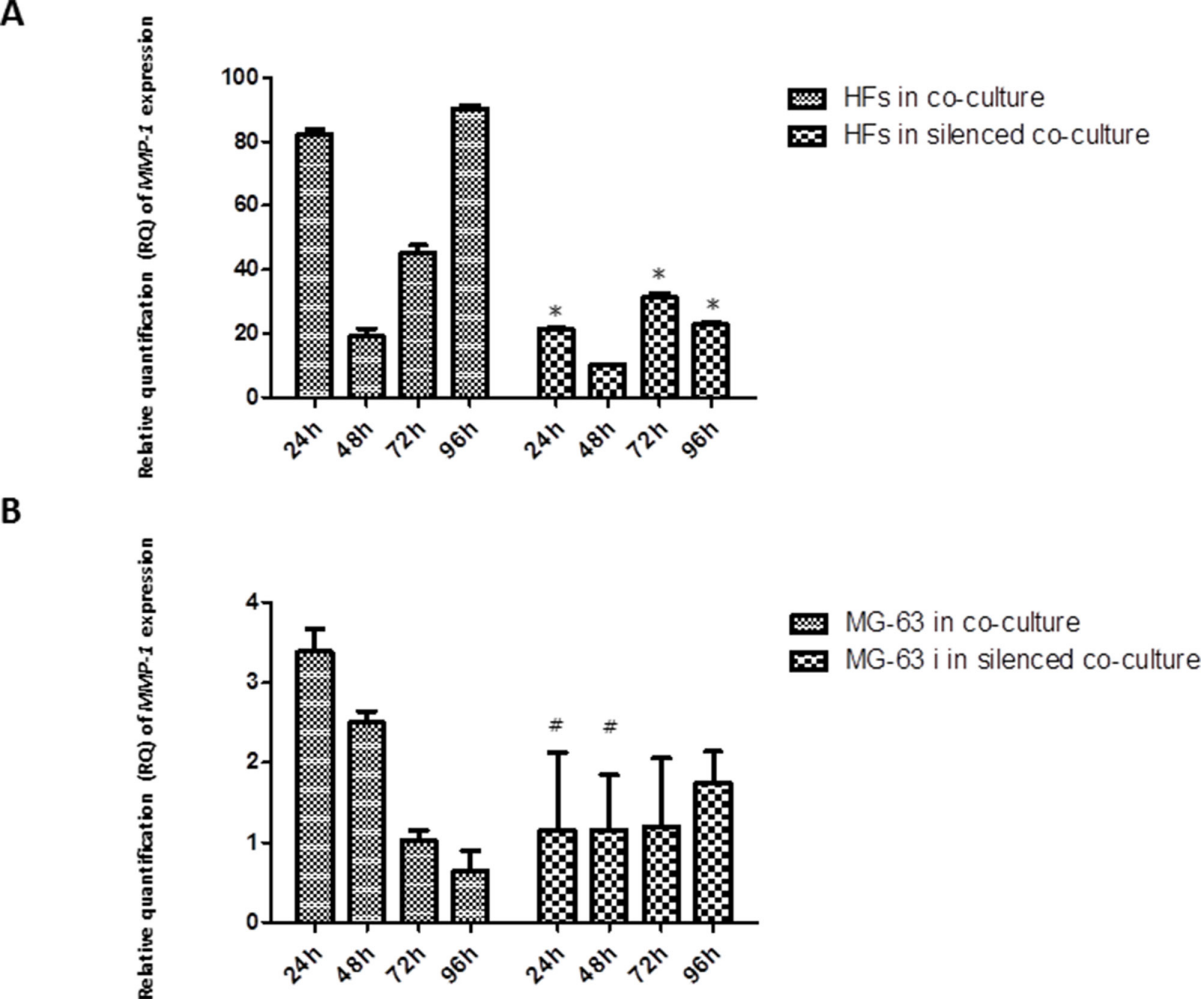
A. and B Real-Time PCR for *MMP-1*. The expression of *MMP-1* was weak in MG-63 cells grown in both co-cultures. In normal co-culture, *MMP-1* was strongly expressed in HFs and increased to a maximum peak at 96 h. In the silenced co-culture, its expression was three-fold lower than in the normal co-culture. * represents a significant difference from HF cells grown in normal co-culture, *P* < 0.05. # represents a significant difference from MG-63 cells grown in normal co-culture, *P* < 0.05.

**Figure 6:**
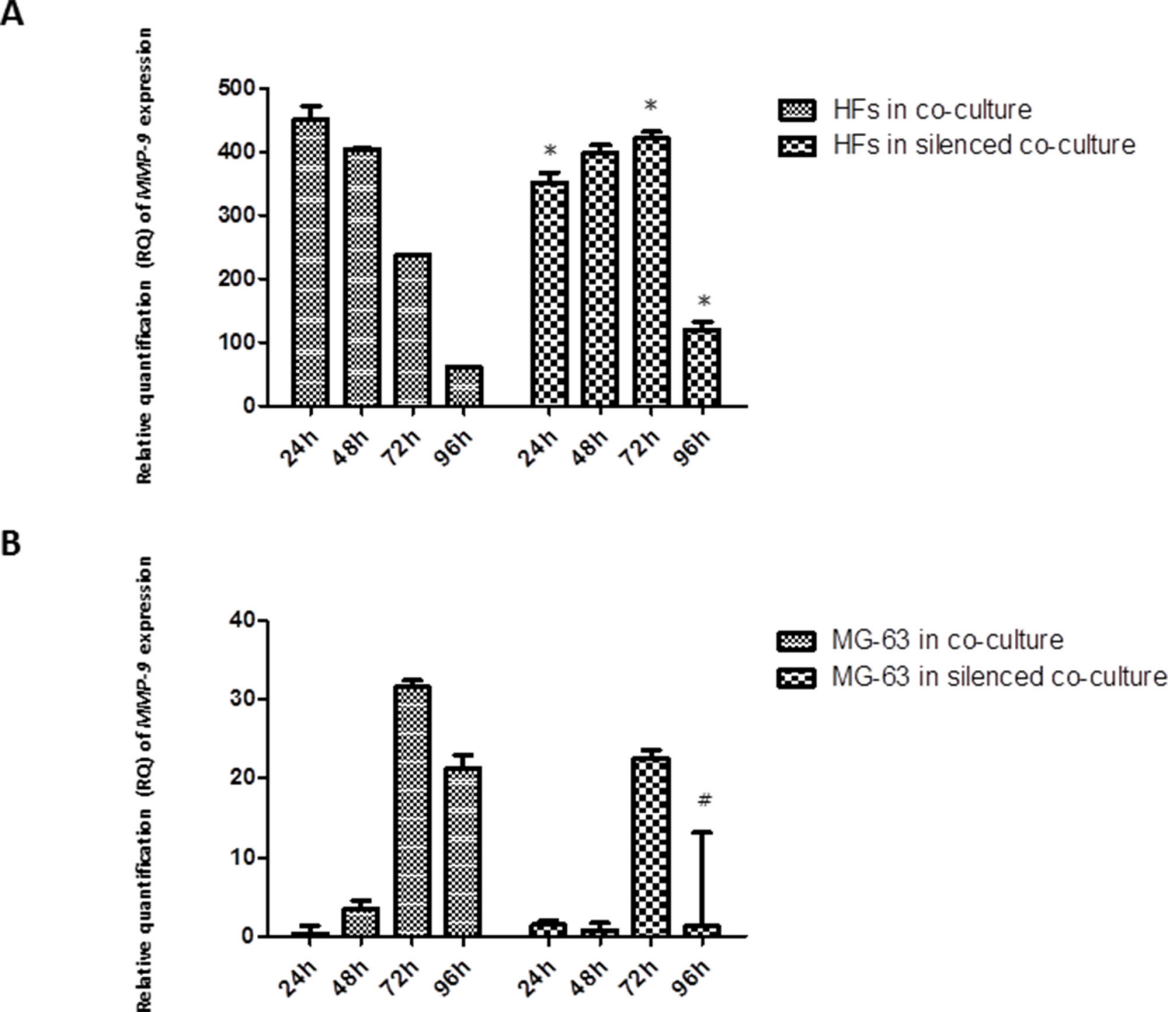
A. and B Real-Time PCR for *MMP-9*. The expression of *MMP-9* tended to decrease steadily as fibroblasts were grown in normal co-culture, whereas in fibroblasts grown in silenced co-culture, it remained fairly constant for at least 72 h, before declining to lower levels by the 96 h time point. The MG-63 cells of both conditions expressed *MMP-9* only weakly. * represents a significant difference from HF cells grown in normal co-culture, *P* < 0.05. # represents a significant difference from MG-63 cells grown in normal co-culture, *P* < 0.05.

*MMP-1* release was observed early in HFs, at 24 h of co-culture with MG-63 cells, reaching the maximum expression at 96 h. In HFs co-cultured with silenced MG-63 cells, the expression of *MMP-1* was significantly less. Both in normally co-cultured MG-63 cells and in silenced co-cultured MG-63 cells, the expression of *MMP-1* was very low (Figure 5). The expression of *MMP-9* decreased from 24 h to 96 h in normally co-cultured HFs and seemed to be constant until 72 h in HFs co-cultured with silenced MG-63 cells. Expression of *MMP-9* in MG-63 cells in both conditions of co-culture was weak (Figure 6).

## DISCUSSION

Investigating the molecular and cellular mechanisms that mediate a sustained wound-healing microenvironment may be important for understanding how the tumor microenvironment promotes tumor progression and metastasis. The complex stromal reaction that occurs during cancer growth involves a persistent secretion of growth factors and cytokines produced by malignant cells. A pre-malignant cellular or tissue injury may stimulate a local inflammatory and acute wound-healing reaction, altering stromal composition and initiating transformed epithelial cell proliferation and expansion. Whereas the signaling pathways responsible for the behavior of malignant carcinoma cells have been the primary focus of cancer research and therapeutic strategies over the last three decades, the reactions of the microenvironment to invading cancer cells are less defined and deserve more attention [3].

In our previous study [23], we analyzed the role of microenvironment in cancer initiation and progression by simulating the coexistence between MG-63 cancer cells and healthy HFs in a monolayer co-culture system. We identified YKL-40 as the main marker for verifying the influence of tumor cells grown in contact with healthy cells, and we found that YKL-40 may have synergistic effects: in HFs, it can act similarly to insulin-like growth factor-1, leading to an inflammatory condition and promoting an up-regulation of *MMP-1* expression, remodeling the ECM. In addition, the expression of *YKL-40* in MG-63 cells correlated with the expression of *VEGF*, suggesting that YKL-40 is an angiogenic factor in tumor cells, as is well-reported in the literature [23].

To establish an adequate *in vitro* model that can better simulate a tumor microenvironment, we used a transwell co-culture system consisting of MG-63 cells and healthy HFs. To confirm the pathological role of YKL-40 during the early stages of tumor progression, we reduced the level of YKL-40 from MG-63 cells with siRNA knockdown. We set up two parallel co-cultures: one with *YKL-40*-silenced MG-63 cells and HFs, and the other with normal MG-63 cells and HFs. Both co-cultures were run for 24 h, 48 h, 72 h, and 96 h to investigate whether the knockdown of YKL-40 influences the tumor microenvironment and the gene expression of several markers. Besides the validation of previously studied *VEGF* and *MMP-1*, we also assessed MMP-9 (another metalloprotease involved in matrix degradation during pathological conditions), TNF-α, and IL-6, which are the molecules responsible for inflammation and the regulation of YKL-40.

The analysis of mRNA expression in a co-culture of HFs and MG-63 cancer cells found a significant trend in the *in vitro* levels of TNF-α. TNF-α is involved in the inflammatory network that contributes to all stages of the malignant process [6]. Many cancer cell lines secrete low picogram-amounts of TNF-α *in vitro* in the absence of inflammatory stimuli, and chronic production of low levels in the tumor microenvironment may cause DNA damage, mediate tumor/stromal cell interactions, and induce a range of MMPs and cytokines that promote tumor development [24]. Our results demonstrate that in MG-63 cells, both in normal co-culture and *YKL-40*-silenced co-culture, the levels of TNF-α are low and constant for all experimental time points. Upon co-culture with tumor cells, the concentration of *TNF-α* mRNA increased significantly in HFs in both types of co-culture, suggesting that YKL-40 does not influence TNF-α expression. TNF-α expression induces a rapid activation of IL-6 in tissue affected by inflammation [7], which explains the significant increase in the expression of *IL-6* in fibroblasts under both co-culture conditions. Again, the silencing of *YKL-40* did not appear to affect the expression of *IL-6*, similar to *TNF-α*; however, there are studies that found that IL-6 and TNF-α are the major regulators during acute inflammation and most likely also regulate the expression of *YKL-40* [14]. Nielsen *et al*. demonstrated that, *in vitro*, increasing the concentration of IL-6 to a higher level than that of TNF-α stimulates the release of YKL-40, suggesting a link between inflammation and increased levels of YKL-40, which agrees with our results. In our study, *YKL-40*, normally expressed only by MG-63 cells, had increasing expression in HFs, confirming our hypothesis about its possible pathological roles during the development of different types of human cancer: association with the angiogenic switch and remodeling of the ECM [23]. With YKL-40 recently identified as a potent angiogenic factor [25], we expected that the siRNA knockdown of YKL-40 would directly affect expression of *VEGF*. In our study, *VEGF* expression was low at all time points in both the co-cultured silenced MG-63 cells and HFs, but expression increased in HFs and MG-63 cells grown in normal co-culture, a result that correlates with the increase in *YKL-40* expression in the same cells.

Our results regarding metalloproteases suggest that in a simulated tumor microenvironment, the HFs represent the main source of MMP production, and they are expressed at significant levels. MMPs are essential for tumor growth, invasion, and metastasis [26]. MMPs do not only originate from the malignant compartment of the tumor, but also from benign cells such as fibroblasts [27], especially fibroblasts growing near tumor cells. These so-called tumor-associated fibroblasts are able to stimulate tumor cell motility and invasion [28]. Both MMP-1 and MMP-9 are up-regulated by TNF-α. In our study, the increase in YKL-40 released by co-cultured fibroblasts seems responsible for the increase in *MMP-1* expression, more so than *MMP-9* expression. Confirming this, in fibroblasts co-cultured with YKL-40-silenced MG-63 cells, the expression of *MMP-1* is lower, not only compared to the expression observed in fibroblasts of the normal co-culture, but also compared to *MMP-9*, which was expressed at high levels in fibroblasts of both types of co-culture. YKL-40 was shown to bind important components of the cartilage ECM, proteoglycans and collagens (the main targets of MMP-1), and to influence their production and assembly [29]. We can therefore hypothesize that, during chronic inflammation, IL-6 stimulates the expression of *YKL-40*, which acts as a regulatory factor in catabolic processes, having a direct influence on *MMP-1* expression, whereas the level of *MMP-9* expression is up-regulated by TNF-α.

## MATERIALS AND METHODS

### Primary culture of HFs

Human gingival tissues were collected from the mid-third of the roots of teeth extracted for orthodontic reasons, following informed consent of the patients. After several washes in PBS, the tissues were cut into small pieces and placed into culture dishes with 1 mL of Dulbecco's Modified Essential Medium (DMEM) (Invitrogen, Carlsbad, CA, USA) supplemented with 10% (v/v) fetal bovine serum (FBS), penicillin (100 mg/mL), and streptomycin (10 mg/mL). The culture medium was changed twice a week. When the HFs were subconfluent (70–80%), they were scraped off using 0.05% trypsin/EDTA (Gibco, Grand Island, NE, USA), washed, and placed into T75 flasks. The cells obtained were cultured at 37°C in a humidified atmosphere of 5% CO_2_. Cells from passages 3 to 10 were utilized for the following experiments.

### MG-63 cell culture

The MG-63 cell line was purchased from ATCC, product CRL-1427 (Manassas, VA, USA) and cultivated in DMEM (Invitrogen) supplemented with 10% (v/v) FBS, penicillin (100 mg/mL), and streptomycin (10 mg/mL). The cells were cultured in T25 flasks (Nunc, Waltham, MA, USA) in a humidified incubator at 37°C in a 5% CO_2_ humidified atmosphere. For passaging, the cells were detached with trypsin/EDTA and subsequently replated.

### siRNA transfection in MG-63 cells

After 4 days in culture, MG-63 cells were transfected with Silencer Select Pre-designed *YKL-40* siRNA. Pre-designed *YKL-40* siRNA (ID s2998) and Silencer Negative Control were purchased from Ambion (Life Technologies, Carlsbad, CA, USA). The day before transfection, cells were seeded at a density of 2.5 × 10^5^ cells/mL in T25 cell culture flasks containing DMEM medium with FBS (10%) and antibiotics (1×). For each transfection (2 mL of plating growth medium), *YKL-40* siRNA (75 pmol) was mixed with 7.5 μL of Lipofectamine RNAiMax Reagent (Life Technologies) in a serum-free medium (MEM, Gibco), incubated for 5 min, and then added to cells. After 3 days, cells were collected and seeded at a density of 2.5 × 10^5^ cells/mL in Transwell Permeable Supports (0.4 μm polyester membrane, 24 mm insert). The viability of MG-63 cells had been verified after the transfection and it was optimal compared to their control. The YKL-40 knockdown efficiency in MG-63 cells was on average of 87%, reaching the 88% between 48 h and 72 h.

### Co-cultures of HFs and MG-63 cells

We performed two types of co-culture, with HFs and MG-63 cells seeded at the same density (2.5  ×  10^5^ cells/mL each). HFs were pre-cultured in 6-well plates, while MG-63 cells and MG-63 cells with silenced *YKL-40* were seeded in 6-well transwell inserts (Nunc). After 24 h, each support was put in the 6-well plates and co-cultured in DMEM supplemented with 10% (v/v) FBS, penicillin (100 mg/mL), and streptomycin (10 mg/mL) for 24 h, 48 h, 72 h, and 96 h. HFs, MG-63 cells, and silenced MG-63 cells were collected and analyzed at the same experimental time points.

### RNA extraction

Total RNA was isolated from HFs and MG-63 cells using Nucleospin RNA II (Macherey-Nagel, Dueren, Germany) and quantified using a NanoDrop ND-1000 UV-vis Spectrophotometer (Thermo Scientific, Wilmington, DE, USA). One microgram of total RNA was reverse transcribed using a high capacity cDNA Reverse Transcription kit (Applied Biosystems, Life Technologies, Monza, Italy).

### Real-time PCR

The analysis of gene expression was carried out by using pre-designed TaqMan^®^ 96-well array plates, code number 4413266, format 8 (Life Technologies), pre-loaded in each reaction well with our choice of inventoried TaqMan^®^ Gene Expression assays (*YKL-40*, *TNF-α*, *IL-6*, *MMP-1*, *MMP-9*, and *VEGF*) and one endogenous control (*GAPDH*) with 10 ng of cDNA per well. The plates were analyzed by quantitative real-time PCR using the 7500 Real-Time PCR machine (Applied Biosystems, Life Technologies, Monza, Italy). All reactions were carried out in a 25 μL reaction volume in triplicate. The comparative 2−ΔΔCt method had been used to evaluate mRNA expression considering as reference the basal expression of mRNAs of HFs and MG-63 cells independently cultured and used as controls. HFs independently cultured were considered as reference for HFs co-cultured in both condition (YKL-40 expressed/ YKL-40 knockdown) and MG-63 cells independently cultured were considered as reference for MG-63s co-cultured in both condition (YKL-40 expressed/ YKL-40 knockdown).

The data are representative of three independent experiments and shown as the average of triplicates  ±  SD. The statistical analysis was carried out using GRAPH PAD PRISM 5.0 software (San Diego, CA, USA) and applying ANOVA and the Dunnett's multiple comparison test. The differences were considered significant at *P* < 0.05.

## CONCLUSIONS

Taken together, these data emphasize the need to further understand the complex molecular networks and crosstalk between different components of the tumor microenvironment and the tumor cells. Knowledge of mechanisms involved in tumor progression, invasiveness, and metastasis could be essential to improving the efficacy of current therapeutic interventions, causing a significant clinical impact.

In the future, combination therapies targeting not only the tumor cells, but also the stroma and associated local and systemic wound-healing processes in the tumor microenvironment will be necessary to successfully treat cancer. Rather than thinking about cancer as a result of individually-transformed cells, considering cancer from a tissue perspective and taking into consideration all the local- and distally-recruited participants will ultimately be the future in treating the disease, and in understanding how tumor cells and the microenvironment respond, adapt, and evolve.

## Abbreviations

HFs: Human fibroblasts
ECM: Extracellular matrix
TNF alpha: Tumor necrosis factor alpha
IL-6: interleukin 6
YKL-40: Human cartilage glycoprotein–39
MMP1 and MMP-9: Matrix metalloprotease 1 and matrix metalloprotease 9
MMPs: Matrix metalloproteinases
VEGF: Vascular Endothelial Growth Factor.

## References

[1] Kidd S, Spaeth E, Watson K, Burks J, Lu H, Klopp A, Andreeff M, Marini FC (2012). Origins of the tumor microenvironment: quantitative assessment of adipose-derived and bone marrow-derived stroma. PLoS One.

[2] C Rodrigues-Lisoni F, Peitl P, Vidotto A, Polachini GM, Maniglia JV, Carmona-Raphe J, Cunha BR, Henrique T, Souza CF, Teixeira RAP, Fukuyama EE, Michaluart P, De Carvalho MB, Oliani SM (2010). Genomics and proteomics approaches to the study of cancer-stroma interactions 2,. Head and Neck Genome Project BMC Medical Genomics.

[3] Kuperwasser C (2010). The tumor stromal microenvironment as modulator of malignant behavior. J Mammary Gland Biol Neoplasia.

[4] Balkwill F (2006). TNF-alpha in promotion and progression of cancer. Cancer Metastasis Rev.

[5] Whiteside T (2008). The tumor microenvironment and its role in promoting tumor growth. Oncogene.

[6] Balkwill F (2009). Tumour necrosis factor and cancer. Nat Rev Cancer.

[7] Lippitz BE (2013). Cytokine patterns in patients with cancer: a systematic review. The Lancet Oncology.

[8] Landskron G, De la Fuente M, Thuwajit P, Thuwajit C, Hermoso MA (2014). Chronic Inflammation and Cytokines in the Tumor Microenvironment. Journal of Immunology Research.

[9] Zarogoulidis P, Tsakiridis K, Karapantzou C, Lampaki S, Pitsiou IKG, Papaiwannou A (2015). Use of Proteins as Biomarkers and Their Role in Carcinogenesis. Journal of Cancer.

[10] Johansen JS, Jensen BV, Anne Roslind, Nielsen D, Price PA (2006). Serum YKL-40, A New Prognostic Biomarker in Cancer Patients?. Cancer Epidemiol Biomarkers Prev.

[11] Basset P, Bellocq JP, Wolf C, Stoll I, Hutin P, Limacher JM, Podhajcer OL, Chenard MP, Rio MC, Chambon P (1990). A novel metalloproteinase gene specifically expressed in stromal cells of breast carcinomas. Nature.

[12] Grégoire M, Lieubeau B (1995). The role of fibroblasts in tumor behavior. Cancer Metastasis Rev.

[13] Rønnov-Jessen L, Petersen OW, Bissell MJ (1996). Cellular changes involved in conversion of normal to malignant breast: importance of the stromal reaction. Physiol Rev.

[14] Nielsen AR, Plomgaard P, Krabbe KS, Johansen JS, Pedersen BK (2011). IL-6, but not TNF-a, increases plasma YKL-40 in human subjects. Cytokine.

[15] Riabov V, Gudima A, Wang N, Mickley A, Orekhov A, Kzhyshkowska J (2014). Role of tumor associated macrophages in tumor angiogenesis and lymphangiogenesis. Front Physiol.

[16] Coussens LM, Fingleton B, Matrisian LM (2002). Matrix metalloproteinase inhibitors and cancer: trials and tribulations. Science.

[17] Egeblad M, Werb Z (2002). New functions for the matrix metalloproteinases in cancer progression. Nat. Rev. Cancer.

[18] Lockhart AC (2003). Matrix Metalloproteinases, Angiogenesis, and Cancer Commentary: Reduction of Wound Angiogenesis in Patients Treated with BMS-275291, a Broad Spectrum Matrix Metalloproteinase Inhibitor. Clin. Cancer Res.

[19] Farina AR, Reay Mackay A (2014). Gelatinase B/MMP-9 in Tumour Pathogenesis and Progression. Cancers.

[20] Kunisch E, Kinne RW, Alsalameh RJ, Alsalameh S (2014). Pro-inflammatory IL-1beta and/or TNF-alpha up-regulate matrix metalloproteases-1 and -3 mRNA in chondrocyte subpopulations potentially pathogenic in osteoarthritis: in situ hybridization studies on a single cell level. Int J Rheum Dis.

[21] Brzozowa M, Michalski M, Harabin-Słowińska M, Wojnicz R (2014). The role of tumour microenvironment in gastric cancer angiogenesis. Prz Gastroenterol.

[22] Faibish M, Francescone R, Bentley B, Yan W, Shao R (2011). A YKL-40-neutralizing antibody blocks tumor angiogenesis and progression: a potential therapeutic agent in cancers. Mol. Cancer Ther.

[23] Salvatore V, Teti G, Bolzani S, Focaroli S, Durante S, Falconi M (2014). Simulating tumor microenvironment: changes in protein expression in an *in vitro* co-culture system. Cancer Cell Int.

[24] Li B, Vincent A, Cates J, Brantley-Sieders DM, Polk DB, Young PP (2009). Low levels of tumor necrosis factor alpha increase tumor growth by inducing an endothelial phenotype of monocytes recruited to the tumor site. Cancer Res.

[25] Shao R, Hamel K, Petersen L, Cao QJ, Arenas RB, Bigelow C, Bentley B, Yan W (2009). YKL-40, a secreted glycoprotein, promotes tumor angiogenesis. Oncogene.

[26] Hagemann T, Robinson SC, Schulz M, Trümper L, Balkwill FR, Binder C (2004). Enhanced invasiveness of breast cancer cell lines upon co-cultivation with macrophages is due to TNF-α dependent up-regulation of matrix metalloproteases. Carcinogenesis.

[27] Ito A, Nakajima S, Sasaguri Y, Nagase H, Mori Y (1995). Co-culture of human adenocarcinoma MCF-7 cells and human dermal fibroblasts enhances the production of matrix metalloproteinases 1, 2 and 3 in fibroblasts. Br J Cancer.

[28] Cirri P, Chiarugi P (2011). Cancer associated fibroblasts: the dark side of the coin. Am J Cancer Res.

[29] Väänänen T, Koskinen A, Paukkeri EL, Hämäläinen M, Moilanen T, Moilanen E, Vuolteenaho K (2014). YKL-40 as a novel factor associated with inflammation and catabolic mechanisms in osteoarthritic joints. Mediators Inflamm.

